# Association between glycated hemoglobin and hematological parameters in patients with type 2 diabetes mellitus: a cross-sectional study

**DOI:** 10.1016/j.clinsp.2026.101122

**Published:** 2026-09-09

**Authors:** Sami G. Almalki, Yahya Madkhali

**Affiliations:** Department of Medical Laboratory Sciences, College of Applied Medical Sciences, Majmaah University, Majmaah, 11952, Saudi Arabia

**Keywords:** HbA1c, Anemia, Inflammation, MCHC, Red blood cell indices, Type 2 diabetes mellitus, Glycemic control, Sex differences

## Abstract

**Background:**

Glycosylated hemoglobin (HbA1c) is a critical biomarker for evaluating long-term glycemic state and diagnosing diabetes mellitus, although hematological abnormalities can affect its clinical interpretation. This study aimed to determine the association of HbA1c with hematological parameters among patients with T2DM; assess associations after adjusting for age and sex; investigate sex-specific differences; and evaluate prevalence of anemia in different glycemic control categories.

**Methods:**

This cross-sectional study evaluated clinical data from 1,278 patients with confirmed T2DM (HbA1c ≥ 6.5%; 726 females, 552 males) aged ≥ 30 years collected between January 2020 and December 2021. Analyses included correlations, age- and sex-adjusted linear regression, interaction testing, analysis of variance, logistic regression, and Holm correction.

**Results:**

The mean HbA1c was 8.56 ± 1.80%. Sex-stratified analysis indicated a sex-dependent reversal in the HbA1c-Hct association, with a positive correlation in females (r = 0.106) and a negative correlation in males (r = -0.110); the interaction remained significant after correction (p = 0.035). MCHC was the hematological parameter most strongly associated with HbA1c overall (r = 0.101, adjusted p = 0.002) and remained significant after adjusting for age and sex (β = 0.095, 95% CI 0.034–0.156; adjusted p = 0.014). In males, HbA1c was inversely associated with MCV (r = −0.140, adjusted p = 0.011). The prevalence of anemia was 22.9% and was significantly higher in females than males (27.7% vs. 16.7%, p < 0.001); HbA1c was not associated with anemia (OR = 0.96, 95% CI 0.89–1.04).

**Conclusions:**

These associations were very weak and require prospective confirmation.

## Highlights

• HbA1c was associated with selected red blood cell indices in T2DM.
• MCHC showed the most consistent adjusted association with HbA1c.
• HbA1c-hematocrit associations differed between female and male patients.
• Anemia prevalence varied significantly across glycemic control categories.
• Findings support cautious HbA1c interpretation with hematological abnormalities.

## Introduction

Diabetes mellitus constitutes a major global health challenge. According to the International Diabetes Federation, over 530 million adults are currently living with diabetes worldwide.^1^ Glycated Hemoglobin (HbA1c) has become the cornerstone of glycemic assessment reflecting average blood glucose concentrations over the preceding 8–12 weeks and serving as a diagnostic criterion and a therapeutic target in diabetes management.^2^^,^^3^

Despite its widespread clinical utility, HbA1c is influenced by glycemia and hematological factors. Because HbA1c represents the proportion of glycated hemoglobin relative to total Hemoglobin (Hb), conditions affecting Hb concentration, such as Red Blood Cell (RBC) turnover or erythrocyte lifespan, may alter HbA1c values independently of prevailing glucose levels.^4^^,^^5^ Iron deficiency anemia is associated with spuriously elevated HbA1c, whereas hemolysis and increased erythropoiesis typically lower HbA1c.^6^, ^7^, ^8^, ^9^ A recent systematic review confirmed that iron deficiency, with or without overt anemia, is associated with significantly higher HbA1c values compared with iron-replete controls.^10^ These findings have important clinical implications, as misinterpretation of HbA1c in the presence of hematological abnormalities may result in inappropriate therapeutic decisions.^11^

Anemia is a common comorbidity among patients with Type 2 Diabetes Mellitus (T2DM), with pooled global prevalence estimates ranging from 27%–35%.^12^^,^^13^ Although anemia and HbA1c interaction have been extensively investigated in non-diabetic populations,^14^^,^^15^ fewer studies have examined these relationships specifically in patients with diabetes. Thus, the clinical focus shifts from diagnosis to treatment. Furthermore, the significance of specific RBC indices, especially Mean Corpuscular Hemoglobin Concentration (MCHC), as correlates of HbA1c in diabetic populations remains underexplored.^16^^,^^17^ Additionally, sex-specific differences in these associations have received limited attention, despite well-established sex-related variations in hematological parameters and in the physiological determinants of HbA1c.^18^^,^^19^ Chronic low-grade inflammation is also common in T2DM and may become more pronounced in patients with diabetic complications.^20^, ^21^, ^22^, ^23^ Several erythrocyte indices obtained from routine blood counts have also been studied as nonspecific markers of inflammation in inflammatory, autoimmune, and gastrointestinal diseases.^24^, ^25^, ^26^, ^27^ This provides further reason to examine routine erythrocyte parameters in patients with T2DM.

Therefore, this study aimed to (1) assess the correlation between HbA1c and hematological parameters in a large cohort of patients with confirmed T2DM; (2) identify which RBC indices remain independently associated with HbA1c after controlling for age and sex; (3) formally investigate sex-specific differences in these associations; and (4) delineate the prevalence and pattern of anemia across categories of glycemic control.

## Materials and methods

### Study design and setting

This single-center, cross-sectional study analyzed routinely collected clinical and laboratory data from patients with confirmed T2DM who attended the King Khaled Hospital in Majmaah City, Saudi Arabia, between January 2020 and December 2021.

### Study population

Consecutive adults with a clinician-documented diagnosis of T2DM were eligible for inclusion if they had an HbA1c value ≥ 6.5%, in accordance with American Diabetes Association (ADA) diagnostic criteria, and underwent complete blood count testing concurrently with HbA1c measurement during the study period. Participants with known hemoglobinopathies, chronic kidney disease, active malignancy, pregnancy, or blood transfusion during the prior 3 months were excluded, as these conditions may affect hematological parameters and interfere with HbA1c interpretation. To limit the potential for misclassification of diabetes subtype, analyses were confined to those aged ≥ 30 years. This age restriction was an eligibility criterion and was not used as the basis for the T2DM diagnosis. Although the restriction was based on the epidemiological pattern that T2DM mainly occurs after 30 years, it may exclude young-onset T2DM, and autoantibody and C-peptide data were unavailable. This age exclusion removed 131 patients (44 aged < 18 years and 87 aged 18–29 years), resulting in a final analytical cohort of 1,278 adults with T2DM (726 females and 552 males, aged 30–98 years).

### Data collection and laboratory measurements

Demographic characteristics (age and sex) and laboratory data were retrieved from electronic health records and the laboratory information system. To ensure temporal concordance, we obtained values of HbA1c and complete blood count during the same clinical visit. All laboratory analyses were performed according to established internal quality control and external quality assurance procedures. HbA1c was determined using the VARIANT II TURBO (Bio-Rad) analyzer, based on the principles of high-performance liquid chromatography. No values were missing for the variables included in the analysis.

### Definitions

Anemia was defined based on World Health Organization criteria as Hb < 13.0 g/dL in males and < 12.0 g/dL in females.^28^ Anemia was further classified morphologically according to mean corpuscular volume (MCV) as microcytic (< 80 fL), normocytic (80–100 fL), or macrocytic (> 100 fL). Glycemic control was defined as good (HbA1c ≤ 7.0%), moderate (7.1–9.0%), or poor (> 9.0%), in accordance with ADA treatment targets.^3^

### Statistical analysis

Continuous variables are expressed as mean and standard deviation, whereas categorical variables are expressed as frequency and percentage. Assumptions of distribution were checked using histograms and Q–Q plots before analysis, and parametric and nonparametric methods were applied where appropriate. Bivariate associations between HbA1c and hematological parameters were examined using Pearson and Spearman correlation coefficients. Age- and sex-adjusted linear regression with HC3 robust standard errors was performed to examine whether the observed correlations remained after controlling for these factors. Because several RBC indices are closely related, each was examined in a separate adjusted model. Ridge regression was also used as a sensitivity analysis. One-way analysis of variance (ANOVA) was used to compare differences among glycemic control groups. Homogeneity of variance was tested using Levene's test, and post hoc comparisons used Tukey’s test or, when assumptions were not met, Welch analysis with Games–Howell comparisons. Chi-square tests were conducted to examine anemia prevalence, and logistic regression assessed its association with HbA1c after adjustment for age and sex. Additionally, analyses were stratified by sex, and formal interaction terms were tested. Linear and quadratic contrasts and restricted cubic splines assessed non-linear patterns. Holm correction was applied within each family of multiple tests. The analytical dataset had no missing values. A two-sided p < 0.05 was considered statistically significant. All analyses were performed using IBM SPSS Statistics (version 25.0; IBM Corp., Armonk, NY, USA). Reporting was guided by the STROBE statement for observational studies.^29^

### Ethical considerations

Ethical approval of the study was received from the Ministry of Health in Saudi Arabia (Institutional Review Board Approval Number: 22-485E; approval date: 2 November 2022). The study was conducted in accordance with the principles of the Declaration of Helsinki, and all patient information was anonymized and handled confidentially during the study.

## Results

### Baseline characteristics of the study population

This analysis comprised 1,278 patients with confirmed T2DM aged ≥ 30 years. Of these, 726 (56.8%) were females and 552 (43.2%) were males. The mean age of the participants was 53.96 ± 12.93 years, and the mean HbA1c was 8.56 ± 1.80% (range 6.60–16.80%). According to glycemic control categories, 326 patients (25.5%) had good glycemic control, 517 patients (40.5%) had moderate control, and 435 patients (34.0%) had poor control. No data were missing for the variables considered. Table 1 shows overall and sex-stratified baseline demographic and hematological characteristics.

**Table 1:** Baseline demographic, glycemic, and hematological characteristics overall and by sex.

Variable	Overall (n = 1,278)	Female (n = 726)	Male (n = 552)	p-value
Age (years), mean ± SD	53.96 ± 12.93	52.91 ± 13.07	55.36 ± 12.63	0.001
HbA1c (%), mean ± SD	8.56 ± 1.80	8.46 ± 1.75	8.69 ± 1.86	0.026
Good control, n (%)	326 (25.5)	197 (27.1)	129 (23.4)	0.077
Moderate control, n (%)	517 (40.5)	300 (41.3)	217 (39.3)	-
Poor control, n (%)	435 (34.0)	229 (31.5)	206 (37.3)	-
Hb (g/dL), mean ± SD	13.54 ± 1.98	12.73 ± 1.67	14.61 ± 1.83	<0.001
RBC (×10¹²/L), mean ± SD	4.86 ± 0.62	4.66 ± 0.53	5.13 ± 0.63	<0.001
Hct (%), mean ± SD	41.85 ± 5.54	39.74 ± 4.78	44.62 ± 5.25	<0.001
MCV (fL), mean ± SD	86.13 ± 6.64	85.32 ± 7.19	87.19 ± 5.67	<0.001
MCH (pg), mean ± SD	27.87 ± 2.88	27.31 ± 3.04	28.61 ± 2.48	<0.001
MCHC (g/dL), mean ± SD	32.36 ± 1.71	32.01 ± 1.64	32.81 ± 1.69	<0.001

Values are mean ± SD or n (%). Continuous-variable p values are Holm-adjusted; glycemic-category and anemia p values are from chi-square tests. All participants had a clinician-documented diagnosis of T2DM and HbA1c ≥6.5% at inclusion. Abbreviations are as defined in the text.

### Correlations of HbA1c with hematological parameters

Among the hematological parameters studied, MCHC showed the highest correlation with HbA1c in bivariate analyses (Pearson r = 0.101, Holm-adjusted p = 0.002; Spearman ρ = 0.098, Holm-adjusted p = 0.003). Hb and RBC were weakly positively correlated with HbA1c at the conventional level of significance but not after Holm correction. However, hematocrit (Hct), MCV, and mean corpuscular hemoglobin (MCH) were not significantly associated with HbA1c in the combined-sex analysis. The stronger Spearman coefficient for age (ρ = 0.189) relative to the Pearson coefficient (r = 0.138) suggested a potential non-linear relationship between age and HbA1c,^30^ which was confirmed by spline analysis (p = 0.0014).

**Fig. 1:**
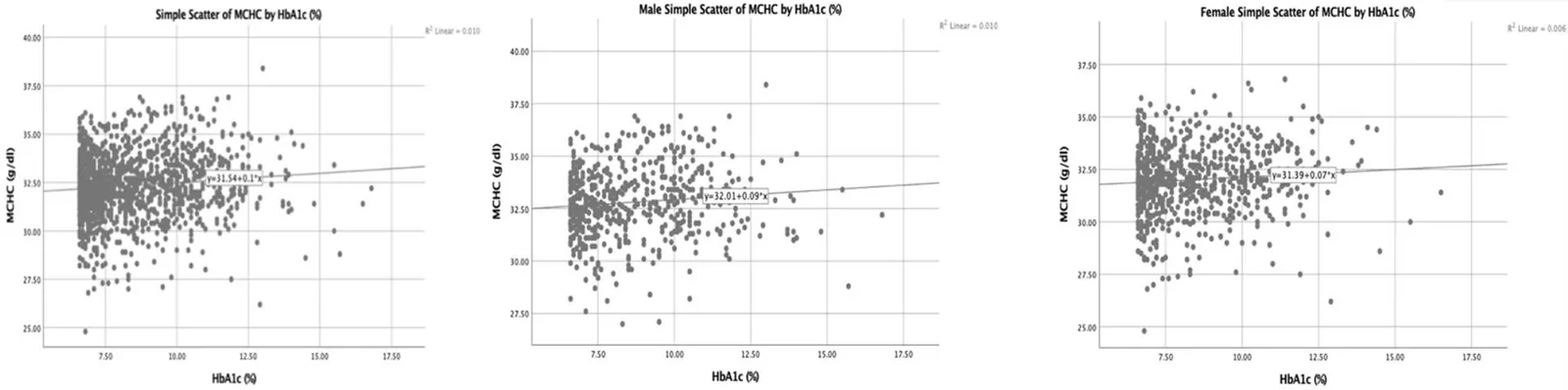
**Association between HbA1c and mean corpuscular hemoglobin concentration (MCHC) in the overall cohort and by sex.** Scatter plots with linear regression lines illustrate the relationship between HbA1c and MCHC in (left) the overall study population, (center) male patients, and (right) female patients. A positive but very weak association was observed in all three panels. In the overall cohort, MCHC remained associated with HbA1c after age and sex adjustment (standardized β = 0.090; Holm-adjusted p = 0.014).

After adjustment for age and sex using partial correlation analysis, MCHC remained significantly associated with HbA1c (partial r = 0.088, p = 0.002). RBC and MCV also remained significantly associated but in opposite directions (RBC partial r = 0.078, p = 0.005; MCV partial r = −0.074, p = 0.008). Hb was no longer significantly associated after adjustment (partial r = 0.050, p = 0.077), whereas Hct and MCH showed no significant associations (data not shown). Although statistically significant, the magnitude of the MCHC association was modest and should not be interpreted as indicating a large clinical effect. Fig. 1 depicts the relationship between HbA1c and MCHC in the overall cohort and by sex. In age- and sex-adjusted linear regression, MCHC remained associated with HbA1c (β = 0.095 percentage points per 1 g/dL, 95% CI 0.034–0.156; Holm-adjusted p = 0.014), whereas RBC and MCV did not remain significant after correction (both adjusted p = 0.056). All effect sizes were very small, and the MCHC model explained little HbA1c variation (adjusted R² = 0.027) (Table 2).

**Table 2:** Overall correlations and age- and sex-adjusted linear regression associations with HbA1c.

Variable	Pearson r (95% CI)	Holm p	Spearman ρ	Holm p	Adjusted β (95% CI)	Std. β	Holm p
Hb	0.064 (0.009, 0.119)	0.086	0.071	0.054	0.051 (−0.007, 0.108)	0.056	0.255
RBC	0.068 (0.013, 0.122)	0.078	0.068	0.061	0.249 (0.056, 0.442)	0.086	0.056
Hct	0.031 (−0.023, 0.086)	0.521	0.037	0.380	0.006 (−0.015, 0.027)	0.018	0.944
MCV	−0.039 (−0.094, 0.016)	0.487	−0.061	0.088	−0.020 (−0.036, −0.005)	−0.075	0.056
MCH	0.012 (−0.043, 0.067)	0.669	0.007	0.806	−0.014 (−0.051, 0.023)	−0.022	0.944
MCHC	0.101 (0.046, 0.154)	0.002	0.098	0.003	0.095 (0.034, 0.156)	0.090	0.014

Each adjusted model included age, sex, and one hematological parameter, with HbA1c as the outcome and HC3 robust standard errors. Holm correction was applied separately to each analysis family. β values represent change in HbA1c percentage points per unit increase. Std. β, standardized coefficient.

### Sex-stratified correlations

Sex-stratified analyses showed a clear sex-dependent reversal of the association between HbA1c and several hematological parameters (Table 3). Hct exhibited the most significant pattern, which was positively correlated with HbA1c in females (r = 0.106, p = 0.004) but negatively correlated in males (r = −0.110, p = 0.010). We also observed a similar directionality pattern of directionality for Hb, with a significant positive correlation in females (r = 0.131, p < 0.001) and a non-significant negative tendency in males (r = −0.064, p = 0.131). Among females (n = 726), HbA1c was positively correlated with Hb, RBC (r = 0.113, p = 0.002), Hct, and MCHC (r = 0.078, p = 0.036). Whereas among males (n = 552), HbA1c exhibited significant negative correlations with Hct and MCV (r = −0.140, p < 0.001), and a positive correlation with MCHC (r = 0.101, p = 0.017). After Holm correction, the associations for female Hb-HbA1c, female RBC–HbA1c, female Hct–HbA1c, and male MCV-HbA1c remained significant, whereas the remaining sex-specific associations were considered nominally significant only. Formal interaction testing confirmed a sex difference only for Hct (interaction β = 0.336, 95% CI 0.097–0.575; Holm-adjusted p = 0.035); the Hb and MCV interactions did not survive correction. These results extend previous observations of sex-dependent effects of Hb on HbA1c to a cohort with confirmed diabetes and suggest that Hct may provide a more sensitive indicator of this sex-dependent reversal than Hb alone.^30^^,^^31^

**Table 3:** Sex-stratified correlations and formal sex-by-parameter interaction tests.

Variable	Female r (95% CI)	Holm p	Male r (95% CI)	Holm p	Interaction β (95% CI)	Holm p
Hb	0.131 (0.059, 0.202)	0.005	−0.064 (−0.147, 0.019)	0.523	0.290 (0.051, 0.529)	0.088
RBC	0.113 (0.041, 0.185)	0.022	−0.020 (−0.104, 0.063)	1.000	0.195 (−0.044, 0.434)	0.331
Hct	0.106 (0.034, 0.178)	0.037	−0.110 (−0.191, −0.027)	0.079	0.336 (0.097, 0.575)	0.035
MCV	0.009 (−0.064, 0.082)	1.000	−0.140 (−0.221, −0.057)	0.011	0.291 (0.048, 0.533)	0.088
MCH	0.043 (−0.029, 0.116)	0.728	−0.071 (−0.154, 0.013)	0.479	0.191 (−0.051, 0.433)	0.331
MCHC	0.078 (0.005, 0.150)	0.215	0.101 (0.018, 0.183)	0.120	−0.085 (−0.297, 0.127)	0.432

Female n = 726; male n = 552. Correlation and interaction p values were Holm-adjusted. Interaction models were age-adjusted and tested the female–male difference in HbA1c slope. Only the Hct interaction remained significant after correction.

### Hematological parameters across glycemic control categories

**Fig. 2:**
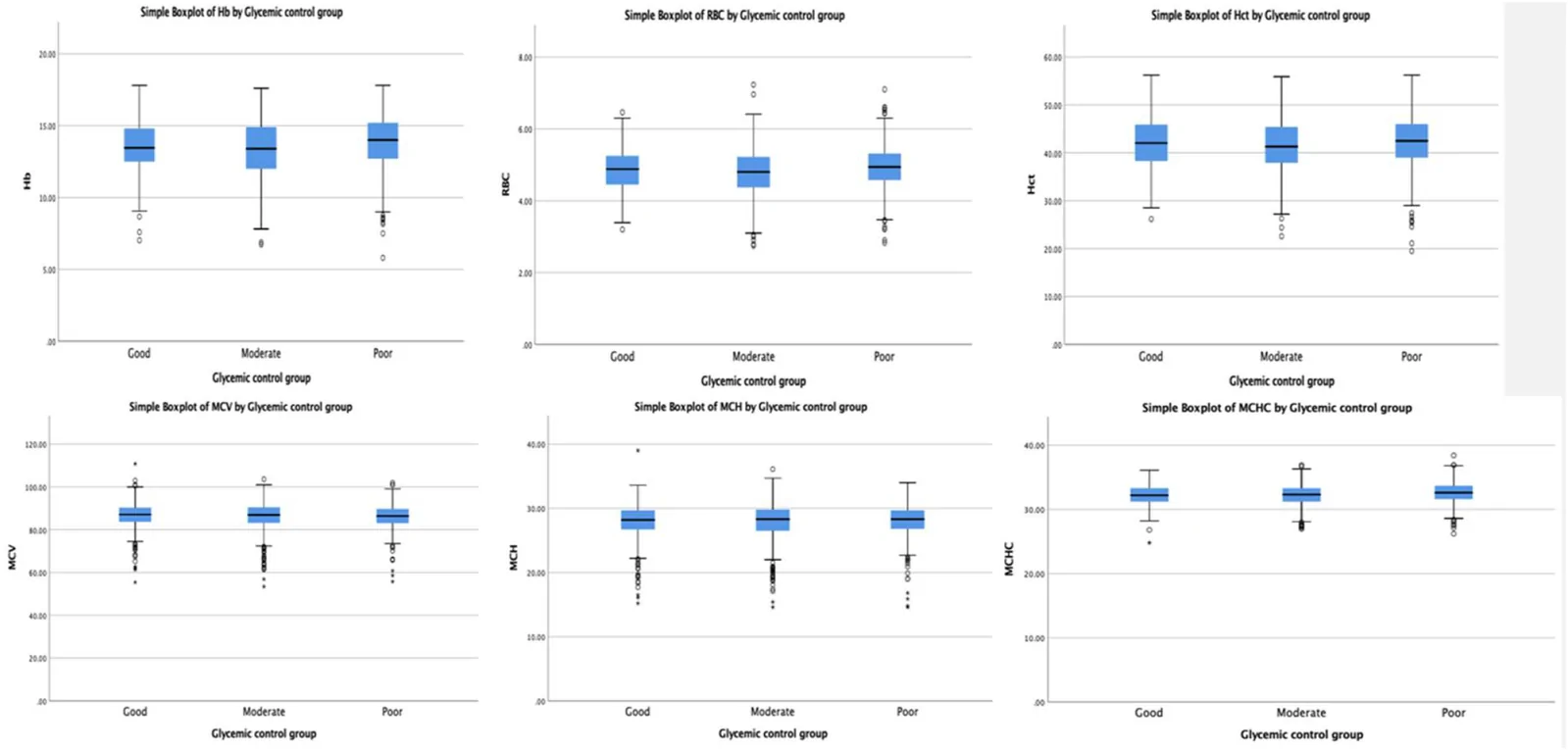
**Distribution of hematological parameters across glycemic control categories.** Box plots compare hemoglobin (Hb), red blood cell count (RBC), hematocrit (Hct), mean corpuscular volume (MCV), mean corpuscular hemoglobin (MCH), and mean corpuscular hemoglobin concentration (MCHC) among patients with good (HbA1c ≤ 7.0%), moderate (7.1–9.0%), and poor (> 9.0%) glycemic control. Boxes indicate the interquartile range, and the horizontal line indicates the median; whiskers are 1.5 × IQR. Quadratic category contrasts were significant for Hb, RBC, and Hct, but continuous spline analyses did not confirm non-linearity for these indices. MCHC differed across categories, although the effect size was small.

One-way ANOVA demonstrated differences across glycemic control categories for Hb (F = 7.83, p < 0.001), RBC (F = 6.36, p = 0.002), Hct (F = 4.27, p = 0.014), and MCHC (F = 8.67, p < 0.001). The lowest mean values for Hb, RBC, and Hct were observed in the moderate control group, whereas the highest values were observed in the poor control group, indicating a non-linear pattern rather than a monotonic gradient. No significant differences were observed across glycemic control categories for MCV and MCH. Conversely, MCHC showed a stepwise increase across categories from good to poor glycemic control (32.22 ± 1.63, 32.21 ± 1.71, and 32.63 ± 1.74 g/dL, respectively; p < 0.001). This pattern is consistent with a graded cross-sectional association rather than evidence of a causal dose–response relationship (Table 4). After Holm correction, differences remained for Hb, RBC, Hct, and MCHC, but effect sizes were small (ω² = 0.005–0.012). Quadratic contrasts showed differences across categories for Hb, RBC, and Hct. However, spline analyses using the continuous data did not confirm these non-linear patterns, suggesting that the findings may depend on the selected cut-points. Fig. 2 depicts the distributions of hematological parameters across glycemic control categories.

**Table 4:** Hematological parameters across glycemic-control categories.

Variable	Good (n = 326)	Moderate (n = 517)	Poor (n = 435)	Holm p	ω²	Quadratic Holm p
Hb	13.56 ± 1.86	13.30 ± 2.03	13.81 ± 1.98	0.002	0.011	0.004
RBC	4.86 ± 0.58	4.79 ± 0.63	4.94 ± 0.63	0.007	0.008	0.034
Hct	42.06 ± 5.24	41.31 ± 5.60	42.32 ± 5.66	0.043	0.005	0.034
MCV	86.50 ± 7.07	86.06 ± 6.98	85.93 ± 5.84	0.878	0.000	0.483
MCH	27.88 ± 3.02	27.76 ± 2.98	28.00 ± 2.65	0.916	0.000	0.445
MCHC	32.22 ± 1.64	32.21 ± 1.71	32.63 ± 1.74	0.001	0.012	0.093

Values are mean ± SD. Omnibus p values and quadratic-contrast p values were Holm-adjusted. All effect sizes were small. Significant pairwise comparisons were moderate versus poor for Hb, RBC, and Hct and good versus poor and moderate versus poor for MCHC.

### Anemia prevalence and distribution

**Fig. 3:**
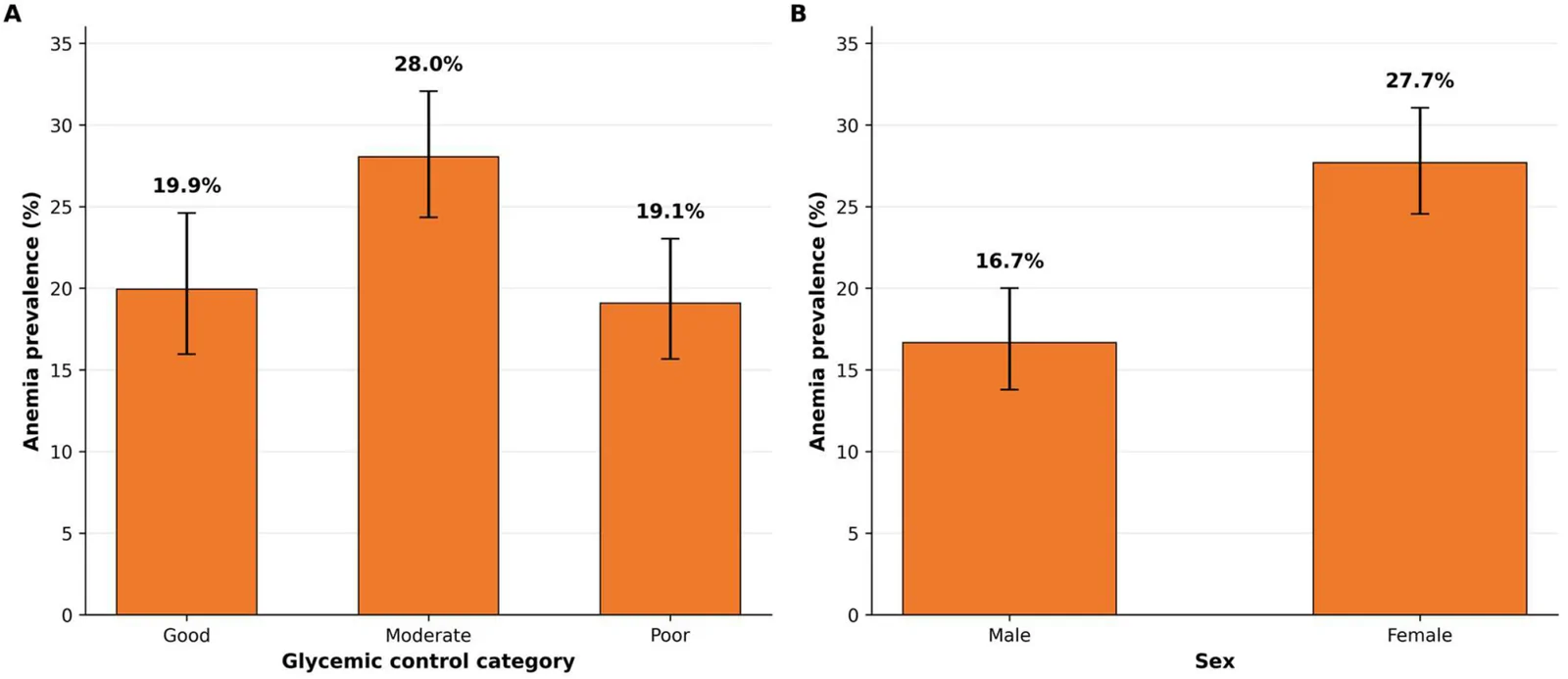
**Prevalence of anemia according to glycemic control category and sex.** Anemia prevalence with 95% confidence intervals is shown according to glycemic-control category and sex. Anemia was defined according to WHO criteria as hemoglobin < 13.0 g/dL in males and < 12.0 g/dL in females. Prevalence differed across glycemic-control categories (p = 0.002), with the highest prevalence in the moderate group, and was higher in females than males (27.7% vs. 16.7%, p < 0.001).

Using World Health Organization (WHO) criteria, 293 patients (22.9%) were classified as anemic. Anemia was more prevalent among females than males (27.7% vs. 16.7%, p < 0.001), consistent with global anemia epidemiology and previous reports from diabetic populations.^32^ Anemia prevalence also varied significantly across glycemic control categories (χ² = 12.96, df = 2, p = 0.0015), with the highest prevalence observed in the moderate control group (28.0%), compared with the good (19.9%) and poor (19.1%) control groups (Fig. 3). Holm-adjusted pairwise comparisons confirmed differences between moderate and good control and between moderate and poor control, but not between good and poor control.

Despite these differences in anemia prevalence, mean HbA1c values did not differ significantly between anemic and non-anemic patients (8.47 ± 1.79% vs. 8.61 ± 1.80%, p = 0.238). In age- and sex-adjusted logistic regression, continuous HbA1c was not associated with anemia (OR 0.96, 95% CI 0.89–1.04; p = 0.325). Moderate control was associated with higher odds of anemia than good control (OR 1.55, 95% CI 1.11–2.17), whereas poor control was not. However, specific types of anemia may still affect the interpretation of HbA1c (Table 5).

**Table 5:** Logistic regression models for anemia.

Model	Predictor	Odds ratio	95% CI	p-value
Continuous HbA1c	Age (per year)	1.008	0.997–1.019	0.140
Continuous HbA1c	Female vs. male	1.940	1.481–2.542	<0.001
Continuous HbA1c	HbA1c (per 1%)	0.962	0.889–1.040	0.325
Glycemic categories	Age (per year)	1.007	0.996–1.018	0.195
Glycemic categories	Female vs. male	1.941	1.479–2.548	<0.001
Glycemic categories	Moderate vs. good	1.552	1.111–2.169	0.010
Glycemic categories	Poor vs. good	0.949	0.659–1.367	0.778

Models were adjusted for age and sex. The continuous model evaluates HbA1c per 1-percentage-point increase; the categorical model uses good glycemic control as the reference group.

## Discussion

This cross-sectional study of patients with type 2 diabetes mellitus expands current understanding of the hematological factors that may influence HbA1c interpretation. The principal finding was a clear sex-dependent divergence in the association between HbA1c and Hct, characterized by positive correlations in females and inverse correlations in males. Hb demonstrated a similar, although less symmetric pattern, with a positive correlation observed in females and a non-significant inverse correlation in males. After adjustment for age and sex, MCHC emerged as the most consistent RBC correlate of HbA1c. Additionally, HbA1c was inversely associated with MCV among males, suggesting the presence of smaller erythrocytes at higher levels of glycemic dysregulation. However, the magnitude of the MCHC-HbA1c association was modest, and the findings should be interpreted primarily as hypothesis-generating rather than as a basis for immediate changes in clinical practice. All effect sizes were very small.

The consistency of the MCHC–HbA1c association is biologically plausible. HbA1c reflects glycated Hb relative to total Hb, whereas MCHC approximates the intracellular Hb concentration.^4^^,^^5^^,^^33^ Accordingly, a positive correlation between these parameters may partially reflect inter-individual variation in erythrocyte Hb concentration or related erythrocyte characteristics. A 1 g/dL increase in MCHC corresponded to only a 0.095 percentage point increase in HbA1c, and the model explained approximately 3% of HbA1c variation. Nevertheless, the present dataset does not permit distinction between direct biological mechanisms and residual confounding; therefore, mechanistic interpretations should remain cautious.

Previous studies have yielded heterogeneous results. One investigation in patients with T2DM reported positive associations between MCHC and red cell distribution width with inadequate glycemic control,^16^ whereas another identified an inverse relationship between MCH and HbA1c-defined dysglycemia.^31^ In non-diabetic populations, inverse MCHC-HbA1c relationships have been reported, particularly in the context of iron deficiency.^6^^,^^7^ Collectively, these findings suggest that the direction and magnitude of RBC-HbA1c associations may vary according to population characteristics, anemia profiles, and the specific clinical setting in which HbA1c is interpreted.

The HbA1c-Hct association exhibited a significant sex-dependent reversal that appeared more prominent and symmetrical than the Hb-HbA1c relation. Among males, higher HbA1c was additionally associated with lower MCV values, indicating smaller red cells at poorer glycemic control. Although such sex-dependent patterns have been reported less frequently than the broader association between anemia and HbA1c, they are supported by previous literature. Bae et al. demonstrated that sex-specific differences in Hb concentration influence HbA1c interpretation in non-anemic Korean populations,^18^ and Sakamoto et al. reported similar sex-dependent associations in a large cohort of Japanese workers.^19^ Formal interaction testing confirmed the sex difference for Hct only; the MCV interaction did not remain significant after correction.

Several mechanisms may potentially explain these findings, including sex-related differences in iron metabolism, erythropoiesis, hormonal milieu, and metabolic physiology.^34^^,^^35^ However, these mechanisms could not be evaluated directly in this study because iron studies, inflammatory markers, medication data, and other potential relevant covariates were unavailable. Consequently, the observed sex-specific pattern should be regarded as an important descriptive finding that warrants targeted prospective investigation.

The higher prevalence of anemia in the moderate glycemic control group is an important cross-sectional result; however, it needs to be interpreted with caution. Possible causes include variation in anemia subtypes, unassessed iron status, or clinical selection criteria affecting the distribution within glycemic categories. Formal quadratic contrasts supported the category based pattern, but continuous analyses did not confirm non-linearity for Hb, RBC, or Hct. The apparent U-shaped distribution may therefore depend partly on the selected cut-points and should be considered descriptive rather than mechanistic.

Notably, despite significant variation in anemia prevalence across glycemic categories, mean HbA1c values did not differ significantly between anemic and non-anemic patients (8.47% vs. 8.61%, p = 0.238). Age- and sex-adjusted logistic regression similarly showed no association between continuous HbA1c and anemia. However, this does not demonstrate that anemia has no effect on HbA1c interpretation. Nevertheless, more detailed phenotyping of anemia, including iron status, vitamin B12, folate, subtype, and severity, might identify clinically relevant subgroups not captured by this binary classification.^36^

The present findings have several practical implications that should be interpreted in the context of the very small effect sizes and cross-sectional design. First, the relatively high prevalence of anemia reinforces the importance of routine clinical awareness regarding hematological comorbidity in patients with T2DM.^12^^,^^13^^,^^32^^,^^37^ Second, the consistent but weak association between MCHC and HbA1c suggests that RBC-related factors may be relevant when HbA1c values appear discordant with the broader clinical picture, although the current evidence is insufficient to recommend MCHC as an independent adjunctive biomarker. Third, the observed sex-specific divergence in the HbA1c-Hct association warrants further investigation but does not support sex-specific HbA1c thresholds. In patients with substantial hematological disturbance, alternative glycemic markers of glycemic status, such as glycated albumin, may therefore remain clinically useful.^38^

This study has several notable strengths. First, it includes a relatively large, clinically well-characterized cohort of patients with confirmed T2DM, allowing assessment of hematological correlates of HbA1c within a clinically relevant diabetic population rather than in predominantly non-diabetic or diagnostic settings. Second, the analytical approach incorporated complementary parametric and nonparametric correlation analyses, age- and sex-adjusted regression, formal interaction testing, sex-stratified analyses, and correction for multiple testing, thereby enhancing the robustness and transparency of the findings. Furthermore, the identification of a formal sex interaction for Hct represents a potentially novel observation that may inform future mechanistic investigation.

This study has several limitations. First, the cross-sectional design precludes causal inference. Second, the observed effect sizes were modest, limiting immediate clinical applicability. Third, data on iron studies, fasting glucose or continuous glucose monitoring data, diabetes duration, glucose-lowering medications, comorbidities, inflammatory markers, renal function across the full spectrum, and body mass index were unavailable and therefore could not be included in adjusted analyses. Furthermore, this single-center study limits external generalizability; therefore, there is a need for prospective multicenter studies with broad clinical characterization. The study also lacked data on smoking, alcohol consumption, liver disease, erythropoietin therapy, vitamin B12, folate, and diabetic complications; residual confounding is therefore likely. Restriction to HbA1c ≥ 6.5% introduced spectrum bias, and anemia could not be phenotyped by cause or subtype. The cross-sectional design cannot distinguish analytical interference with HbA1c from biological association. Although T2DM was clinician-documented, autoantibody and C-peptide data were unavailable, and the age restriction may have excluded young-onset T2DM.

## Conclusions

This cross-sectional study of 1,278 patients with T2DM demonstrated that the HbA1c and hematological parameter relationship differed significantly by sex, with a formal sex interaction for Hct. After adjustment for age and sex, MCHC demonstrated the most consistent overall association with HbA1c, whereas MCV was inversely associated with HbA1c in males. Our results support cautious interpretation of HbA1c in the context of hematological abnormalities and the need for prospective investigation, although all associations were very weak and do not establish causal direction or analytical interference.

## Declaration of generative AI and AI-assisted technologies

During the preparation of this work, the authors used AI-assisted tools for language editing, manuscript organisation, and references formatting. All AI-generated content was reviewed, edited, and verified by the authors, who take full responsibility for the accuracy, scientific integrity, and originality of the published work, in accordance with applicable journal policies on AI use.

## Institutional review board statement

Ethical approval for this study was obtained from the Ministry of Health, Saudi Arabia (Institutional Review Board Approval Number: 22-485E; approval date: 2 November 2022). This study was conducted in accordance with the principles of the Declaration of Helsinki.

## Informed consent statement

Not applicable. (An IRB certificate can be provided upon request.)

## Funding

This study was supported by the 10.13039/501100011665Deanship of Scientific Research at Majmaah University, Saudi Arabia (project number: R-2026-369).

## CRediT authorship contribution statement

**Sami G. Almalki:** Conceptualization, Methodology, Formal analysis, Investigation, Data curation, Writing – original draft, Writing – review & editing, Project administration. **Yahya Madkhali:** Conceptualization, Methodology, Formal analysis, Investigation, Data curation, Writing – original draft, Writing – review & editing, Supervision.

## Declaration of competing interest

The authors declare no conflicts of interest.

## Data Availability

All data generated or analyzed during this study are included in this published article.
